# Professional Egotism

**Published:** 1864-11

**Authors:** A. Lawrence


THE
DENTAL REGISTER OF THE WEST.

VOL. XVIII.] NOVEMBER, 1864. [No. 11.

### Original Essays and Communications.

### PROFESSIONAL EGOTISM.

Read before the Merrimac Valley Dental Association, Nov. 3, 1864.

BY. A. LAWRENCE, M. D.

"Self love never yet could look on truth,
But with blear'd brains; sleek flattery and she
Are twin-born sisters, and so mix their eyes,
As if you sever one, the other dies."

A reasonable respect for self is indispensable to the well-being and success of every man.

Too little or two much is not beneficial in any proper sense, for in either case we regard the unfortunate victim as without the pale of propriety.

One who has come to the conclusion, sans effort, that he has no qualifications, no genius or talents worth respecting, either by himself or others, generally acts in truthful, undisguised unison with his convictions, and is at any rate an honest man—though a fool.

As a dentist, he will be gross in his habits and bungling in his practice.

He will take no pains to improve either his head or his hand, for he justly thinks himself a mean scamp, worth neither the trouble nor the expense.

Egotists, like other men, are possessed of different degrees of intelligence and education; but with much of either the evil in question could not exist. Great men are not egotists; per contra, egotists are not great men.

Indigenious nowhere, egotism pervades all communities, and seems to spread with civilization, so that even this country bids fair to rival old Gascony in that for which she was chiefly remarkable. Professional men, of all others, seem most susceptible to the insidious attacks of this dire monster. Clergymen, lawyers, physicians and dentists, are to be found swelling the ranks of the army under Gen. Bombastes, leaving to other professions the prerogative of looking after their own affairs. Let us examine a representative from each class, the ignorant and the somewhat learned of our own.

"An ignorant dentist" is about the worst title a man ever had or can have, but when the profix "egotistical" properly applies, it becomes absolutely disgusting.

Unfortunately our profession is not without such characters. Delighted with the idea of being called "doctor,"—really unqualified to enter any profession—too lazy to pursue any laborious calling—believing dentistry to require very little brains, yet largely remunerative to those fortunately within its ranks, many a man has so far mistaken his true position as to undertake to discharge the delicate and responsible duties devolving upon the dental surgeon. Now the very fact that an ignorant booby is found attempting the practice of dentistry is prima facie evidence of a large amount of concentrated assurance, or egotism; without which he would have foreseen what he had to encounter, and would have entered the profession only after a thorough preparation under the guidance of some able tutor, followed by the endorsement of the faculty of a Dental College. But, ignorant as he is, he thinks himself a genius, needing only a very little "showing" to be superior, or at least equal, to the best.

He scorns the idea of putting himself under tuition for two or three years to learn so little, and thus wasting his time and money in listening to the "stupid efforts of a set of stuck up fellows calling themselves professors," for four or eight months more.

Three months occasional attendance in the office of some kind friend, who has perhaps "fixed" his teeth, is all he needs, and he is ready to "locate."

Of course he goes to the city, where his services are much needed, and where, in all probability, he will be appreciated, as city people know something, dress better, etc., etc.

He takes rooms, and is prepared to render his services to those requiring a *skillful* dentist. He hangs out at the door

"A great uncouth golden tooth,"

and a box in which a few sets of rudely made teeth champ and chatter nervously together as if for a wager. A notice, "masticating done here," would make the thing complete.

He heads his advertisements with a huge wood cut of a set of teeth—tells the public how many years he has been inserting teeth on vulcanized rubber, boasts of the number of "hull" sets he has furnished—enlarges verbosely on his "new machinery and other extensive improvements introduced into his laboratory," as reasons why he can under bid the lowest fees, and informs the dear people that in "consequence of his *long* experience he is warranted in doing their work in the highest style of the art."

How culpable must one be who should have the temerity to perform a good operation in dentistry with a *short* experience, however well qualified!

Our ignorant egotist has other ways of showing his ruling passion besides advertising. His dress and manners too plainly indicate how deep the waters run. Supercillious in the extreme, his blatant conversation, alike disgusting to every one, invariably displays the pronoun of the first person singular at every word. Brutal and bungling in a practice based upon no theory, he vaunts his skill to increase the faith of his "customers." Neither inuendoes nor positive lying about his more worthy competitors for public favor, lack utterance. Their private character is assailed; their public acts called in question—their professional efforts ridiculed and pronounced worthless.

All this, and much more, one who is a nuisance anywhere, but particularly in a profession to which he is a disgrace.

How long will the public tolerate and encourage such fellows?

Leaving, in utter disgust, the boastful character just under consideration, I turn to another, and for some reasons, more tolerable class of egotists, *i. e.*, the educated ones. Of these men comparatively little need be said in derogation, as, unlike the ignorant, they strive to excell, and frequently do. Many of them are splendid operators. They are ambitious and fond of flattery, but their constant tribulation is an apprehension that all the world will fail to accord to their performances an excellence unapproachable by others. Should an operation of equal or even superior merit come under his observation, no acknowledgement of such merit escapes him. Probably, or rather possibly, he will admit that "it looks well," and then sneeringly intimate that "friend Chronos will shortly discourse upon the subject," "that time will tell," etc. Everything he does or says is right; everything done or said by another is wrong, or at least open to serious objection.

He is loud in his denunciation of everybody in the practice, except a few kindred spirits who may chance to reside too far away to come in contact with those "upper ten," who are exclusively *his* friends and patients. These partially educated egotists, however, do not all, nor always, operate well. A gold filling, which in six weeks presents an appearance about it like a dirty finger nail, they strenuously insist is "all right," if done by themselves, as they never, under any circumstances, do any bad work; but if done by another it is a fraud upon the patient. Of course such services as *his* must be paid for, yet he can never quite make the public come up to his notions about fees, and is therefore obliged to accept the miserable pittance of from twenty to one hundred and fifty dollars for filling a tooth, which his superior in everything but egotism, would have done better for half the money, and thought himself well paid.

An extortionate charge for an operation is not *positive* evidence of skill. This class of egotists are great experts in hobby riding—in practicing specialties, or in doing things in some peculiar way,—great in small things,—in using certain materials to the exclusion of all others, and in condemning every instrument or appliance not made after *their* pattern Certain choice spirits have recently appeared, to dazzle the dental world, who insist that the *mallet* is the emblem, par excellence, of superior skill—that those who fill teeth as well or better without it, are no dentist, simply *because* they do not use it. It is mallet, mallet, mallet, ad infinitum.

Now, I have only to say, that if A can fill a tooth better by its use, it is most certainly his duty to employ it; but if B can do better without such an implement, it does not necessarily follow that he is a quack, merely because he does not adopt A's practice. There are many ways in which egotism manifests itself. The M. D. is too apt to look down on the D. D. S. and vice versa, and both ignore him with neither title, while the latter despises both.

This is all wrong. Let charity prevail.

Those with titles should not disgrace them, and those without should aim to merit them.

The ignorant egotist does not know enough to mend the habit, for it is nothing else, and the discerning public understand it; but there is no excuse for the educated man. The public do not understand why a man should "think more highly of himself than he ought to think," and not "soberly," especially as his education would seem to be sufficient to correct any such unbecoming manifestations.

It was said by some ancient genius, that "man was born with a sack on his shoulders, his neighbors faults in front and his own behind."

That was undoubtedly the case, and rationally accounts for most of our complaints and poor opinions of others, while our own imperfections are neither seen nor thought of, except by those about us.

Let us reverse the sack occasionally, that we may to some extent at least, "see ourselves as others see us!"

> "A little learning is a dang'rous thing;
> Drink deep, or taste not the Pierian spring;
> There shallow draughts intoxicate the brain,
> And drinking largely sobers us again."

